# The Heart’s Electromagnetic Field in Emotions, Empathy and Human Connection: Biosensor-Derived Insights into Heart–Brain Axis Mechanisms and a Basis for Novel BioMagnetoTherapies

**DOI:** 10.3390/s26051738

**Published:** 2026-03-09

**Authors:** Andreas Palantzas, Maria Anagnostouli

**Affiliations:** 1 Athens School of Medicine, National and Kapodistrian University of Athens (NKUA), 10679 Athens, Greece; 2 Multiple Sclerosis and Demyelinating Diseases Unit, Center of Expertise for Rare Demyelinating and Autoinflammatory CNS Diseases, 1st Department of Neurology, School of Medicine, National and Kapodistrian University of Athens, NKUA, Aeginition University Hospital, 11528 Athens, Greece

**Keywords:** heart–brainaxis, electromagnetic field, coherence, geomagnetic activity, emotions, empathy, magnetic neuromodulation, multiple sclerosis, AI models, BioMagnetoTherapies

## Abstract

The heart’s electromagnetic field (HEMF) represents the strongest magnetic signal in the human body and has been increasingly associated with processes related to the Heart–Brain Axis (HBA). The present review summarizes its biophysical basis along with current and emerging biosensing technologies. It examines hypotheses regarding interpersonal interactions and interactions with external fields, including geomagnetic activity, and reviews evidence linking the HEMF to autonomic activity and emotional states. It provides an overview of magnetic field-based therapeutics, introduced here as our own term “BioMagnetoTherapies” (BMT), underscoring their common objective of externally inducing, stabilizing or restoring coherence across the HBA. Collectively, it positions cardiac electromagnetic signals as both a measurable marker, key to HBA dynamics and related disorders, as well as a promising target for emerging biosensor- and BioMagneto-Therapeutics.

## 1. Introduction

### 1.1. Heart–Brain Axis Mechanisms

The heart–brain axis (HBA) consists of a complex interplay of neural, immune, molecular, and hormonal mechanisms that generate quantifiable electrophysiological and autonomic signals that can be assessed via electrocardiogram (ECG)- and heart rate variability (HRV)-based biosensing modalities. Central autonomic pathways regulate cardiac activity through sympathetic and parasympathetic outputs while the intrinsic cardiac nervous system, located at the surface of the heart, acts as a hub, integrating these signals. The neuro-immune cardiovascular interface (NICI) along with inflammatory cytokines, extracellular vesicles and the gut-barrier also contribute to heart–brain communication whereas hormonal systems, such as the hypothalamic–pituitaryaxis (HPA) and the renin-angiotensinsystem (RAS) link stress responses to cardiovascular outcomes [1]. Reflex and feedback pathways—including the baroreflex, chemoreflex, and cardio-respiratory coupling—ensure rapid cardiovascular adaptation. Dysregulation of these mechanisms underpins various neurologic and cardiac disorders [2]

### 1.2. Electromagnetic Fields as an Emerging, Measurable Component of the Heart–Brain Axis

Notably, the heart generates its own electromagnetic field (EMF), arising from ionic fluxes during cardiomyocyte depolarization and repolarization [3,4]. It represents the strongest endogenous magnetic signal in the human body, up to 100 times stronger than the brain’s [5,6], and constitutes a detectable biophysical signal associated with cardiac electrophysiology and subsequently, HBA homeostasis. Its proposed role as a mechanism, facilitating emotional and empathic connectivity along with reported associations with autonomic regulation and documented interactions with external fields, supports consideration of EMFs as a measurable biosignal, relevant to heart–brain physiology, and warrants further validation [7,8,9,10,11,12,13,14,15,16,17,18,19].

The present review aims to synthesize the biophysical basis of the HEMF and evaluate emerging biosensing and magnetic-based technologies, positioning it as a measurable biomarker of the HBA and as a potential, novel target for emerging BioMagnetoTherapies (BMT). It further explores proposed interactions with external and interpersonal fields, as well as its relevance to autonomic and emotional regulation.

## 2. The Heart’s Electromagnetic Field (HEMF)

### 2.1. Biophysical Basis of the Heart’s Electromagnetic Signal

**Figure 1:**
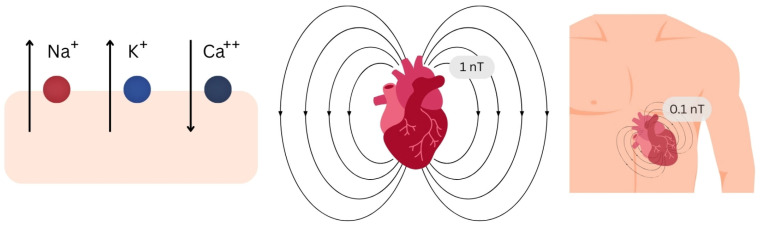
The heart’s electromagnetic field generated by transmembrane ionic fluxes with a strength of 1 nT near the myocardium and 0.1 nT at the chest’s surface.

The heart is the body’s largest bioelectrical source, producing stronger surface potentials than nerves or skeletal muscle [20,21,22]. Its EMF is generated throughout the cardiac cycle and is mainly attributed to transmembrane Na^+^, K^+^, and Ca^2+^ fluxes during depolarization and repolarization, generating time-varying magnetic signals, detectable by sensitive biosensors, in line with Maxwell’s equations [Figure 1] [22,23,24,25,26].

Field strength is ~1 nT near the myocardium, ~0.1 nT at the body surface, and 10–100 pT a few centimeters above the chest—much weaker than the Earth’s field but far stronger than cortical sources [Figure 1] [20,21,22,23,25,26,27].

This field is shaped by conductivity gradients from surrounding thoracic tissues. Lung tissue, due to its high electrical resistivity (~2000 Ω·cm) compared to ventricular muscle (~400 Ω·cm) and blood (~160 Ω·cm), impedes radial EMFs and favors tangential currents [22,27].

Structural features such as cardiac torsion—due to the helical orientation of myocardial fibers—and vortex blood flow patterns, are also speculated to modulate the amplitude and organization of the heart’s electromagnetic field (HEMF) [20,21].

Magnetohydrodynamic and electromagnetic induction effects have also been hypothesized as potential contributors to cardiac biomagnetic signals. In principle, pulsatile ejection of charged particles through the aorta during ventricular repolarization would interact with the magnetic field via the Lorenz force [23,28,29], while electromagnetic induction effects have been proposed to alter membrane potential dynamics, contributing to excitability states and arrhythmic spiral wave activity [24]. However, under physiological conditions where the HEMF has a strength in the order of nanoteslas, such effects are negligible and thus omitted from cardiac tissue models.

### 2.2. Proposed Mechanisms for Magnetic Field Interactions with Biological Systems

The strength of the HEMF, measured at ~1 nT at the level of the heart and at ~0.1 nT at the body surface, represents a significant limitation to the hypothesis that the HEMF may influence biological systems and coherence across the HBA, since such weak magnetic fields are considered biologically irrelevant [20,27]. Though several mechanisms have been proposed for explaining EMF interactions with biological systems, including phase-synchronization [30,31] and ion cylcotron resonance [32], these frameworks do not apply for the heart’s extremely weak magnetic field. The radical pair mechanism and stochastic resonance on the other hand, emerge as two more promising candidates that satisfy this condition.

#### 2.2.1. Radical Pairs

The Radical Pair Mechanism (RPM) proposes that magnetic fields can influence chemical reactions by modulating the quantum spin dynamics of transient radical pairs. Magnetic fields alter the coherent interconversion between singlet and triplet configurations, and because these spin states lead to different reaction outcomes, reaction yields are determined by spin coherence rather than reaction energetics [33,34,35]. In this context, magnetic sensitivity is non-linear, with strongest effects occurring under weak magnetic fields in the microtesla (μT) range [33,36]. As a result, even small magnetic perturbations can produce large changes in reaction yields, explaining how even weak magnetic fields such as the HEMF could exert measurable biologic effects.

While RPM does not directly address the cardiac field or emotional processes, it provides an experimental and theoretical basis that renders the hypothesis regarding biologic systems’ sensitivity to such weak magnetic fields mechanistically plausible. Simplified representation of reaction dynamics and environmental conditions, coherence lifetime constraints along with the need for amplification and coordination across biologic networks represent some important limitations of this mechanism, necessitating further experimental validation [33,34,35,36].

#### 2.2.2. Stochastic Resonance

Stochastic resonance (SR) is a well-established phenomenon where an optimal level of noise described as “stochastic variability”, enhances the impact of weak, sub-threshold signals, such as a weak electromagnetic field, in threshold-based systems. SR allows a weak signal to bias timing, probability and coherence of threshold-crossing events, therefore enabling signal encoding via spike timing, phase alignment, and population-level synchronization [37,38,39].

Cardiac and neural systems satisfy the key conditions required for SR, including nonlinear excitability, intrinsic oscillations, threshold-dependent firing, and substantial background variability arising from ion channel noise, synaptic activity, and autonomic modulation [37,38,39,40].

At a systems level, experimental and computational studies show that SR enhances phase locking, coherence, and synchronization across neuronal populations and sensorimotor networks, promoting transient coordination without requiring strong coupling or direct forcing [37,38,39,40]. Importantly, because the stochastic component supporting SR may be intrinsic or externally applied, these systems are susceptible to external fluctuations such as weak environmental electromagnetic variability [37,38,39].

While no studies have directly examined how this mechanism could be applied to cardiac electromagnetic fields or heart–brain coherence, stochastic resonance provides a biophysically plausible basis for how weak cardiac and environmental electromagnetic variability can influence biological systems. By enabling sub-threshold electromagnetic fluctuations to bias collective dynamics, SR could be relevant to hypotheses in which loss or restoration of heart–brain coherence is associated with detectable electromagnetic signatures, and geomagnetic activity can interact with cardiac electromagnetic and autonomic dynamics. Further research and investigation of these possibilities could lead to significant advances in our understanding regarding the role of weak electromagnetic signals in physiological coordination. Empirical validation of such hypotheses would not only establish the capacity of weak electromagnetic signals to interact with the HEMF, but it would also delineate new pathways through which therapeutic modulation of the HBA could be achieved via externally applied magnetic fields, therefore providing mechanistic targets for novel, BioMagnetoTherapies (BMT) aimed at restoring coherence across the axis.

### 2.3. Measurement Techniques for Cardiac Electromagnetic Signals

Since the first recording of cardiac magnetic signal by Baule and McFee in 1963 using induction coils, the development of Superconducting Quantum Interference Devices (SQUIDs) in the 1970s revolutionized the field by achieving femtotesla (10^−6^ nT) sensitivity, making SQUIDs the golden standard for magnetocardiography (MCG). Various alternatives have been explored for MCG—each with specific strengths and limitations [Table 1] [20,21,23,25,26,27].

Among these, SQUID-based MCG and optically pumped magnetometers (OPMs) emerge as the most promising candidates for advancing hypotheses regarding HEMF dynamics and interactions [38,39]. Due to their femtotesla sensitivity and vector-field reconstruction capability, SQUIDs remain essential tools for validating the foundations of the heart’s electromagnetic structure and coherence [20,21,23,25,26]. Systems like the Neuromag TRIUX (MEGIN Qy, Helsinki, Finland) or the CTF MEG platform (CTF Systems Inc., Coquitlam, BC, Canada) represent established applications while more advanced technologies remain under investigation, such as the nanoscale SQUID-on-Tip (SOT) device developed at the Weizmann Institute (Rehovot, Israel), demonstrating ultra-low flux noise [41,42,43]. OPMs on the other hand, like the QZFM series by QuSpin (QuSpin Inc., Louisville, CO, USA), represent a promising, emerging technology that combines near-SQUID sensitivity with room-temperature operation while offering scalability towards wearable and multi-subject recording [25,26,44,45].

The AM-NMOR OPM-based MEG prototype system, developed at Peking University (Beijing, China), has demonstrated room-temperature operation even in the unshielded Earth’s magneticfield, while platforms such as QuanMag Healthcare’s dual-beam OPM system (QuanMag Healthcare Ltd., Glasgow, UK) and fully wireless OPM-MEG prototypes highlight commercialization pathways toward wearable multi-channel systems [46,47].

Though such advances hold significant promise, practical deployment outside controlled research environments remains technically demanding. Both SQUIDs and OPMs are highly sensitive to ambient magnetic interference, thus requiring magnetically shielded rooms or advanced active noise-cancelation systems to achieve sufficient signal-to-noise ratios for reliable isolation of cardiac magnetic signals, limitations that hinder widespread clinical scalability [23,24,25,26,27].

In parallel, advances in tunnel magnetoresistance (TMR) sensors demonstrate rapid progress towards compact, room-temperature biomagnetic detection. Studies report sub-picotesla sensitivity in miniaturized silicon-compatible architectures that enable real-time MCG and neuronal spike detection without cryogenic cooling. These developments position TMR sensors as emerging practical alternatives to cryogenic SQUID platforms, with potential applications in wearable systems and brain–machine interfaces [48,49,50].

**Table 1:** Comparative overview of measurement techniques for magnetocardiography. (MCG): magnetocardiography; (GMR): giant magnetoresistance; (TMR): tunneling magnetoresistance (SERF): spin-exchange relaxation-free; (SQUIDs): superconducting quantum interference devices; (MRI): magnetic resonance imaging.

Measurement Technique	Advantages	Typical Sensitivity	Detection Range	Commercial Status	Limitations	References
Magnetoelectric sensors	Highly sensitive, low power, promising for portable MCG technologies	1 pT/√Hz to 1 nT/√Hz	1 Hz to several KHz	Primarily academic/prototype stage; not clinically routine	Temperature sensitivity, require shielding, experimental	[25,26,51]
SERF atomic magnetometers	Ultra-sensitive, room-temperature, promising for miniaturization	1–10 fT/√Hz	DC to 10^2^ Hz	Commercial research-grade sensors available; not clinically routine	Require shielding, complex, bulky & expensive, not clinically routine	[25,26,52]
Magnetoresistive sensors (GMR, TMR)	Compact, can detect P-wave and QRS after averaging	100–300 pT/√Hz at 1 Hz	DC to 100 kHz	Commercially available sensors; emerging biomagnetic applications	Sensitive to interference, expensive	[23,25,26,53]
Optically pumped magnetometers	Room-temperature operation, promising for wearable MCG	5–25 fT/√Hz	DC to 100–200 Hz; extendable to 2 kHz	Commercial research systems available; emerging clinical translation	Highly noise-sensitive	[25,26,54]
SQUIDs	Ultrasensitive (femtoteslaresolution), high diagnostic accuracy	0.3–5 fT/√Hz	DC to 100 kHz	Established commercial clinical/research systems (mature platform)	Require cryogenic cooling and shielded rooms	[21,22,23,25,26,55]
Proton precession magnetometers	Portable, useful in MRI enhancement	0.1 nT	0.1–4 Hz	Fully commercial (geophysics/industrial); not used clinically for MCG	Calibration issues and noise	[25,26,56]
Fluxgate magnetometers	Highly sensitive and stable, feasible for MCG	0.05–0.1 nT/√Hz	DC to 1 kHz	Mature/established commercial technology (geophysical applications)	Bulky and power-demanding	[25,26,57]
Hall effect sensors	Small and robust	56–800 pT/√Hz	0.4–4 mT	Fully commercial, mass-market semiconductor technology	Limited sensitivity; require shielding	[25,26,58]
Search coil magnetometers	Cost-effective	0.05–2 pT/√Hz	1 Hz to 10 kHz	Commercial as instrumentation sensors; research use in biomagnetics	Noisy and temperature-sensitive	[25,26,59]
Induction coil magnetometers	Simple, effective near the chest	0.1–0.3 pT/√Hz	Hz to 10 kHz	Commercial instrumentation components; not mainstream clinical MCG	Prone to noise	[23,25,26,27,60]

Beyond direct field measurement, large scale systems, like the Global Coherence Monitoring System, track geomagnetic activity that overlaps with physiological rhythms, providing data on how the HEMF interacts with external magnetic fluctuations [21,25,26,61,62]. Advances in sensing technologies may also be important for closed-loop neuromodulation systems which allow for personalized and continuously adjusted interventions based on electromagnetic or physiological biomarkers [63,64,65]. In a future outlook, implantable magnetoelectric or magneto-inductive sensors represent a noteworthy technology that could enable minimally invasive, long-term monitoring and closed-loop modulation, though such application remains experimental [66,67,68].

### 2.4. Artificial Intelligence in Magnetocardiography

In contemporary MCG research, Artificial Intelligence (AI) applications are becoming an essential tool for noise reduction, disease classification, synthetic data generation and edge computing for wearable devices. Early AI models relied on handcrafted feature extraction, combined with conventional classifiers. Collectively, these studies established feasibility but were constrained by handcrafted features, small datasets, and limited external validation [69,70,71,72,73] [Table 2].

A shift toward end-to-end learning is reflected in a study utilizing a multi-task deep framework integrating multiscale spatio-temporal extractors, graph convolutional networks (GCNs), and transformer components for simultaneous ischemic heart disease diagnosis and localization. By replacing handcrafted pipelines with unified representation learning, this approach enables integrated feature extraction and task coordination within a single model [74].

MCG2Vec advances the field through self-supervised pretraining on raw multi-channel MCG signals, eliminating reliance on engineered features and enabling scalable, data-driven representation learning. The incorporation of Grad-CAM further enhances its contribution by providing physiologically coherent interpretability aligned with ischemic, ventricular, and atrial signal components [75].

Tu et al. [76] demonstrate that classical ensemble methods applied to OPM-MCG-derived repolarization and morphological features can yield strong diagnostic performance, particularly when combined with clinical variables. Their findings highlight the discriminative value of specific magnetic repolarization features (e.g., δAr-PN), reinforcing the biological relevance of MCG-derived features [76].

In this domain, DCBAM represents a promising architecture that integrates deformable convolution with attention mechanisms, therefore enhancing the model’s ability to capture complex signal relationships while preserving efficiency and interpretability via Grad-CAM [77].

Complementarily, SkipDAEformer [78] advances the preprocessing stage by providing robust AI-driven noise suppression, improving signal quality prior to classification and thereby increasing the reliability of downstream MCG diagnostic models.

Despite these advances [75,76,77], common structural constraints persist, including limited external validation, modest dataset scale, absence of calibration analyses, and restricted prospective outcome-based evaluation. Additionally, some frameworks rely on anatomical rather than functional endpoints [75] or default hyperparameter configurations without systematic optimization [76]. While these limitations hinder generalizability and clinical translation, future AI applications could substantially enhance signal quality, feature extraction, data synthesis and diagnostic systems, thereby setting the basis for BioMagnetoTherapeutic strategies that leverage magnetic and autonomic biosignals, not simply as descriptive biomarkers but as actionable targets.

**Table 2:** Comparative overview of Artificial Intelligence applications in magnetocardiography. (ML): machine learning; (MCG): magnetocardiography; (IHD): ischemic heart disease; (BNN): Bayesian neural network; (DK): dynamic kernel self-organizing map; (MLP) (CAD): coronary artery disease; (SVM): support vector machine; (XGBoost): extreme gradient boosting; (AUC): area under the curve; (DL): deep learning (NT-Xent): normalized temperature-scaled cross entropy loss; (LVEF): left ventricular ejection fraction, (AF): atrial fibrillation; (CatBoost): categorical boosting; (OPM): optically pumped magnetometer; (DCBAM): deformable convolutional block attention module; (CNN): convolutional neural network.

Study	Model	Input	Task	Dataset (*n*)	Performance	Limitations
Fenici et al. [69]	Classical ML classifiers	Handcrafted multichannel MCG features	IHD diagnosis	147	Sens 75%, Spec 85%, Acc 80%	Handcrafted features; small dataset
Tantimongcolwat et al. [70]	BNN; DK-SOM	Handcrafted features	IHD detection	125	DK-SOM:Sens 86.2%, Spec. 72.7% BNN: Sens 89.7%, Spec 54.5%	Small dataset; engineered inputs
Huang et al. [71]	Multiple MLP models (M1–M11)	10 predefined MCG parameters	CAD/IHD classification	209	Acc 71.2–90.5% (M10): Sens 89.5%, Spec 89.8% (M11): Sens 90%, Spec 91.4%	Handcrafted features
Tao et al. [72]	SVM-XGBoost	164 T-wave features	IHD detection	574	Acc 94.03% Prec 86.56% AUC = 0.98	Limited validation detail
Han et al. [73]	SVM	Handcrafted features	CAD severity	N/A	Sens 67.0% Spec 88.8% AUC 0.876	Limited validation scale
Tao et al. [74]	Multi-task DL	Averaged MCG cycles; spatio-temporal maps	IHD diagnosis + localization	2.158	(IHD): Sens 83.8%, Spec 85.6%, Acc 84.7% (Localization): Acc 65.3–78.4%	No ECG comparison; limited external validation
Kranz et al. [75]	Self-supervised contrastive encoder MCG2Vec	Raw 64-channel 10 s MCG signals	CAD, LVEF, AF prediction	1732	CAD AUC 0.89; LVEF AUC 0.81; AF AUC 0.77	Single-center; no external validation; no calibration metrics
Tu et al. [76]	Random Forest; CatBoost; SVM; XGBoost	OPM-MCG repolarization + morphology	CAD diagnosis	1513	Heart models: AUC 0.84–0.88 Clinical: AUC 0.62–0.75 Combined: AUC 0.75–0.9	Single-center; default hyperparameters; no external validation
Wang et al. [77]	DCBAM	Hilbert-encoded 36-channel MCG images	CAD classification)	N/A	Acc 93.57%; Sens 92.56%; Spec 94.68%; F1 93.60%;	No broad external validation; preprocessing dependence

### 2.5. Interactions with External Fields and Physiological Implications

#### 2.5.1. Strong Static and Clinical Fields

MRI fields (1.5–3 tesla) induce reversible ECG waveform changes via magnetohydrodynamic effects, that result in altered sinoatrial currents associated with reduced blood flow [23,28,29].

#### 2.5.2. Artificial EMFs

**Figure 2:**
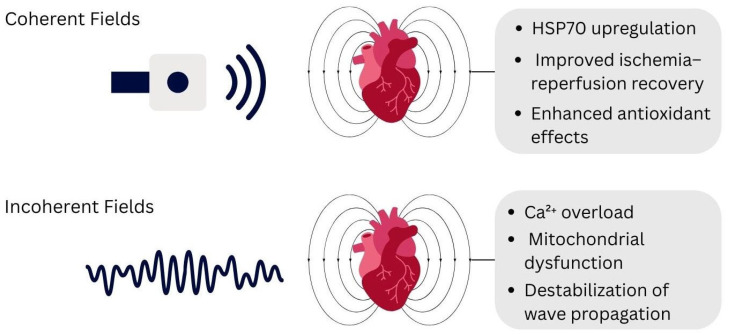
Schematic comparison of cardiac interactions with coherent versus incoherent electromagnetic fields. (HSP70): heat shock protein 70.

Extremely low frequency fields (ELFs) (50/60 Hz) can alter cardiac electrophysiology, producing changes in QTc dynamics, heart rate, and calcium flux [23,29]. Laboratory studies confirm both protective and adverse outcomes depending on frequency and coherence: coherent fields, including pulsed ELFs, upregulate heat shock protein 70 (HSP70), improve ischemia–reperfusion recovery, and enhance antioxidant effects [25,79,80,81] whereas incoherent or intense exposures can worsen injury through Ca^2+^ overload, mitochondrial dysfunction, or destabilization of wave propagation [Figure 2] [24,29,82].

### 2.6. Geomagnetic Interactions

The Earth’s magnetic oscillations, including Schumann and field-line resonances, overlap with cardiovascular and brain rhythms [21,23,61,62,83]. HRV demonstrates sensitivity to solar wind, Schumann power, and geomagnetic disturbances with effects on autonomic balance, blood pressure, and arrhythmia risk [21,61,62,83,84,85,86]. Sensitivity varies among individuals, with arrhythmia patients often showing reduced or negative coupling [54]. Natural geomagnetic oscillations in delta, theta, alpha, and beta frequencies are linked to protective or adaptive effects, while gamma oscillations (32–65 Hz) correlate with higher risk of acute coronary syndromes, especially in women. Geomagnetic disturbances are associated with reduced HRV, infarction, arrhythmias and sudden death, whereas increased Schumann resonance power and favorable solar indices correlate with improved autonomic balance and emotional states [21,61,62,83,84,85,86]. Seasonal geomagnetic variations may modulate these responses [83,84,85].

### 2.7. Interpersonal and Geomagnetic Coupling

The heart’s electromagnetic field may influence other systems, contributing to systemic organization and interpersonal coupling. Electroencephalogram (EEG)–ECG studies show that it influences brain activity [20], while laboratory and population studies suggest that human rhythms may synchronize globally through geomagnetic coupling, indicating a potential mechanism for collective physiological coherence [21,61,62].

Altogether, these findings suggest that external electromagnetic fields—whether artificial or geomagnetic, as well as interpersonal and environmental coupling, may induce measurable changes in cardiac electrophysiology, autonomic balance and HRV—which are core biomarkers and regulatory components of the HBA—thus providing a mechanistic basis through which coherence across the HBA may be influenced.

## 3. Magnetic Field Dynamics in Emotional States and Human Connection

It is suggested that the body’s magnetic field, particularly the heart’s and brain’s, encode and transmit emotional information. Different emotional states generate distinct biomagnetic fields that may reflect the body’s metabolic state [87]. Positive emotions (love, compassion, appreciation) generate coherent HRV patterns, radiating ordered electromagnetic signals, while negative emotions (fear, anger, anxiety) result in incoherent signals and energy loss [87,88,89,90,91]. Among those, fear has the largest bio-field signature [87].

The heart’s magnetic field, being the strongest in the body, can influence nearby nervous systems, enabling heart-to-heart synchronization and physiological coupling [21,90,91,92,93]. EEG–ECG studies confirm cross-individual rhythm synchronization, even without direct contact, and proximity can allow one person’s ECG to register in another’s EEG [87].

Exposure to external magnetic fields can influence emotional responses: geomagnetic storms reduce HRV and increase stress; fields simulating the International Space Station heighten autonomic reactivity to emotional stimuli [94]; low strength, low frequency fields induce measurable ECG changes [95] and exposure to the electromagnetic equivalent of emotional words can alter perception [96].

When individuals are in similar emotional states, their magnetic fields become more coherent and can spread contagiously, influencing others and leading to synchronized responses. Mediated by the earth’s magnetic field, this phenomenon can scale globally, explaining why mass emotional events (e.g., 9/11) are detectable in geomagnetic data [87].

According to theoretical and computational models, billions of brains immersed in Earth’s geomagnetic field may generate a secondary, “transcerebral” field, capable of storing and transmitting collective information, normalizing interneuronal coherence and thus scaling synchronization from neural networks to planetary systems [30,97,98,99].

Collectively, this evidence indicates that magnetic field interactions may constitute an important mechanism mediating the transmission of emotional information, providing new insights into the nature of interpersonal relationships and human connection.

## 4. Empathy

In this context, empathy emerges as a key facilitator of magnetic field interactions that amplifies coherence and connection. Compassion is consistently identified as a strong emotional state, capable of enhancing coherence among individuals and groups, while also strengthening the effects of an individual’s fields on another’s physiology [21,87,88,89]. Nonverbal-compassionate intent has been shown to induce measurable relaxation and HRV changes in recipients [87]. Clinical data report better outcomes when physicians display empathy [21], further confirming the ability of magnetic fields to transmit emotions and intent while also highlighting the importance of empathy in clinical practice [21,88,92].

Although the above observations support the potential implication of the HEMF in empathy, interpersonal coupling, and emotional states, existing evidence is largely observational and derived from small, single-group studies. While quantum-dynamics offer plausible mechanisms for explaining such phenomena, these remain theoretical and lack empirical validation. Therefore, large-scale replication of “heart-to-heart” synchronization experiments represents a necessary and important direction for future research in order to advance such hypotheses and establish their biophysical basis.

## 5. Magnetic Technologies in Sensor-Guided Therapeutic Interventions—BioMagnetoTherapies

Various long-established, newly developed and currently under investigation magnetic field-based therapeutic strategies, introduced here as our own term “BioMagnetoTherapies”, (BMT) have advanced our intervention capabilities and understanding of the HBA and HEMF. In this section we describe some of them.

### 5.1. Transcranial Magnetic Stimulation

**Figure 3:**
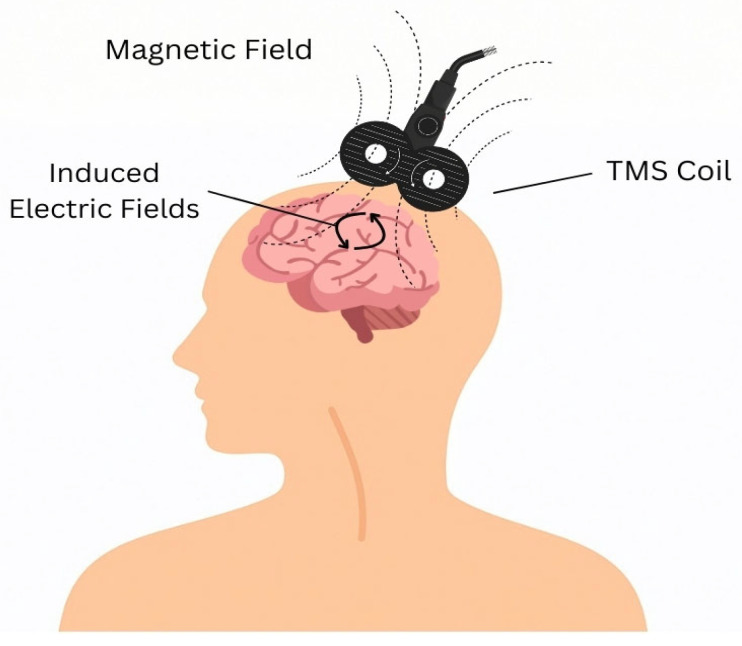
Transcranial magnetic stimulation and cortical electric field induction. (TMS): transcranial magnetic stimulation.

Transcranial magnetic stimulation (TMS) involves the application of controlled magnetic field waveforms to modulate dysfunctional brain circuits [Figure 3]. Deep TMS (dTMS), utilizes H-coils to extend this stimulation to deeper targets, while also offering more favorable safety and tolerability profiles [6,100,101,102,103,104,105]. Accelerated TMS (aTMS/a-iTBS) compresses multiple daily sessions into brief treatment windows to expedite the desired effects.

This approach has been studied extensively in major depressive disorder (MDD), treatment-resistant depression (TRD) and bipolar depression, where MDD and TRD trials targeting predominantly the left dorsolateral prefrontal cortex, show significant antidepressant effects with response and remission rates of 52.2% and 38.2% accordingly [101,104,105,106,107]. Notably, the SAINT/SNT paradigm, shows rapid and significant effects with remission rates up to 78.6% in randomized controlled trials [108,109,110,111]. dTMS has proven effective even in severe and highly resistant patients, reporting durability of up to 12 months [101,102].

Overall, neurophysiological findings suggest normalization of pathological activity in the prefrontal cortex along with potential benefits to cognitive domains such as working memory and attention [112].

In obsessive–compulsive disorder (OCD), stimulation of the medial prefrontal cortex and anterior cingulate cortex produces significant reductions in Yale-brown obsessive-compulsive scores (Y-BOCS) and is associated with improved error-monitoring processes [102,113,114,115].

In multiple sclerosis (MS), TMS targeting motor, prefrontal, and cerebellar regions demonstrates improvements in spasticity, gait, fatigue, and cognitive function [116,117]. Single- and paired-pulse paradigms consistently reveal slowed corticospinal conduction and altered cortical excitability correlating with disability severity and clinical impairment and thus supporting both therapeutic and biomarker potential despite current methodological limitations [118].

In migraines, especially with aura, single-pulse TMS studies report significant reductions in monthly headache days, sustained up to 12 months in difficult-to-treat cohorts [107,108,109,110,111,119,120,121,122], while results for chronic migraine remain inconclusive [117,122].

dTMS protocols are under investigation for substance use disorders, including nicotine, alcohol, and cocaine, with studies demonstrating reductions in craving and consumption, particularly with high-frequency protocols and cue-reactivity procedures [123,124,125].

Results from preliminary research show promise for dTMS and aTMS protocols in other disorders, including schizophrenia [100,101,119,126], post-traumatic stress disorder (PTSD), Parkinson’s disease, obesity [101,127,128,129], OCD, postpartum depression, post-stroke hemiparesis, addictions, Alzheimer’s disease and mild cognitive impairment [119,129]; however, further research is required to confirm these findings.

TMS is also promising in the context of cardiovascular autonomic modulation where it has been shown to enhance vagal activity, improve HRV and cause minor heart rate reductions [11,12,15]. Dorsolateral prefrontal cortex targeting is best for achieving these effects, with both excitatory and inhibitory protocols restoring autonomic balance in healthy individuals and diseased populations, including depression, schizophrenia, PTSD, obesity, bulimia nervosa, chronic pain, and spinal cord injury [11,12].

Novel approaches such as Neuro-Cardiac-Guided TMS and heart–brain coupling further highlight the ability of TMS to engage fronto–vagal circuits in a site- and frequency-specific manner, offering a framework for personalized treatments [13,14]. Clinically, TMS has produced improvements in autonomic regulation in psychiatric disorders and demonstrated promise in stress-related and metabolic conditions, though variability in cardiovascular responses and rare vasovagal reactions highlight the need for personalized dosing and careful monitoring [11,12,130,131].

### 5.2. Magnetic Seizure Therapy

Magnetic seizure therapy (MST) utilizes high-frequency magnetic fields to induce therapeutic seizures, offering more targeted stimulation and reduced spread to medial temporal structures compared to electroconvulsive therapy (ECT). Across studies, MST shows robust antidepressant effects with shorter reorientation times (≈4–9 min) and fewer cognitive adverse effects [132,133,134,135,136,137,138]. Meta-analyses confirm significant overall symptom reduction, while randomized control trials indicate no clear difference to ECT. Reports also indicate benefits in treatment-resistant depression, suicidality, and cognitive outcomes [135,136,137,138], with ongoing large-scale trials such as CREST-MST expected to confirm non-inferiority to right unilateral ultra-brief ECT [138].

Beyond depression, preliminary data on MST show beneficial effects in schizophrenia, bipolar depression, OCD, and borderline personality disorder with suicidality [134,137,139].

Prefrontal, limbic and cingulate networks, which are the main targets in both TMS and MST, represent key components of the Central Autonomic Network (CAN) which refers to a set of cortical and subcortical structures through which the ANS mediates central control over the heart. Through normalization of activity in regions of the CAN, these interventions may rebalance sympathetic and parasympathetic outflow leading to improved HRV and stabilized bidirectional heart–brain signaling. Given that HRV is defined as the key biomarker of HBA integrity, such autonomic rebalancing could reflect partial restoration of functionality and coherence across the HBA [2].

It is important to note that MST, as well as TMS, utilize magnetic fields in the range of teslas and therefore do not reflect or resemble the interactions mediated by the HEMF, which is many orders of magnitude weaker (0.1–1 nT) [23,24,25,26]. Instead, they illustrate the ability of magnetic fields to therapeutically modulate human physiology and neural function, underscore the broader relevance of magnetism in biologic systems and highlight the need for further research to advance even our understanding of subtle, but potentially important, electromagnetic interactions, such as those originating from the heart.

### 5.3. Low-Field and Static Magnetic Fields

Low-frequency magnetic stimulation (LFMS) and static magnetic fields (SMFs) have shown therapeutic potential across neurological and psychiatric disorders. SMFs have shown anti-tumor effects in glioblastoma models via inducing apoptosis, inhibiting angiogenesis and modulating epidermal growth factor receptor (EGFR) and Ca^2+^ pathways [140,141].

In tinnitus models, LFMS (1 Hz auditory cortex) reduced tinnitus perception, likely through long-term depression, while higher-intensity protocols (20 Hz) were also beneficial, likely via spread to auditory cortex [142].

In depression, LFMS produced rapid mood elevation in bipolar and major depression, sometimes immediate, sometimes cumulative, with greater effects in unmedicated patients [142,143,144,145]. Animal studies reported beneficial effects in stress models, while in depression, they demonstrated increased BDNF and dentate gyrus neurogenesis, suggesting a distinct, long-lasting antidepressant mechanism [142]. Clinical trials confirmed that low-frequency, right-prefrontal, repetitive TMS (1 Hz) is antidepressant and preserves cognitive function.

In brain injury and stroke, LFMS reduced inflammation, oxidative stress and apoptosis while upregulating neurotrophic and axon-growth genes, though functional recovery was inconsistent [142].

Findings across Parkinson’s disease, autism and mechanistic studies suggest that LFMS may influence motor, cognitive and neuroplastic processes; however, such evidence is preliminary, inconsistent and highly dependent on stimulation site, protocol and disease context [145,146,147].

Across all paradigms, modulating neuroplastic, inflammatory and oxidative processes that are key to autonomic integrity could provide a hypothetical basis through which LFMS may contribute to restoration of autonomic regulation and in turn coherence across the HBA.

### 5.4. Pulsed Electromagnetic Field Therapy

Pulsed electromagnetic field (PEMF) therapy has demonstrated cardioprotective and neuroprotective potential via enhancing cell survival, reducing inflammation and fibrosis and promoting vascular repair and neuroplasticity [7,8,9,10]. In preclinical models of myocardial infarction and ischemia–reperfusion injury, PEMF improved cardiac function, limited remodeling and enhanced angiogenesis [7,8,9,148]. Beyond cardiovascular applications, PEMF has shown benefits in cerebral ischemia where it reduces infarct size, supports neuronal survival and enhances plasticity while early clinical trials in stroke patients indicate safety, tolerability, and possible lesion reduction, though findings remain inconclusive [10].

### 5.5. Magnetic-Based Therapies

Beyond the interventions presented above, magnetic fields are utilized in a wide range of therapeutic strategies.

Remote magnetic navigation (RMN) has emerged as a safe and effective alternative to manual catheter navigation in cardiac ablation procedures [128,149,150,151,152,153]. Across various arrhythmias including atrial fibrillation, ventricular tachycardia, supraventricular tachycardia and focal atrial tachycardia, RMN achieves high acute success rates compared to manual approaches while reducing fluoroscopy times, radiation exposure and overall complications [150,151,152]. Such benefits are mainly attributed to atraumatic catheter design and improved navigation stability which lower risks of perforation and tamponade [131,152].

At a larger scale, magnetic field-based technologies are transforming endovascular and neurovascular interventions [153,154,155,156,157,158,159,160]. Soft robots, steerable catheters, and magnetic microbots allow non-traumatic vascular navigation, targeted drug or clot therapies, and on-demand embolization [153,155,156,157,158,159,160] with MRI-guided magnetic navigation enhancing visualization and safety [17,155,156]. Early pilots confirm feasibility, though issues of device visibility, retrieval, chronic biocompatibility, and workflow integration require further investigation [153,155,156,157,158,159,160].

## 6. Conclusions

The HBA consists of a complex interplay of numerous, well-established mechanisms with biomarkers, such as heart rate variability, effectively reflecting autonomic balance [2]. This review integrates electromagnetic signaling into this framework by incorporating the HEMF as a complementary and measurable component of the bidirectional cardiac–neural system.

The generation of the HEMF arises from well-established electrophysiological processes and is consistent with classical electromagnetic theory. Advances in biosensing technologies have allowed for robust technological validation through advanced MCG techniques, providing objective confirmation of the detectability and quantification of these signals and reinforcing their biological reality and measurable nature [23,24,25,26].

Although its nanotesla-scale strength (0.1–1 nT) poses a significant limitation for conventional biological interpretation, stochastic resonance and the radical pair mechanism offer plausible theoretical frameworks through which weak magnetic fields could exert measurable effects under specific conditions [35,36,37,38,39]. At present, evidence linking cardiac electromagnetic activity to autonomic regulation, emotional states, and geomagnetic variability remains largely observational and does not establish causality [20,21,61,62].

Collectively, positioning coherence across the HBA as a measurable component provides a structured foundation for integrating biosensing technologies and magnetic-based therapeutic strategies within a unified framework.

## 7. Future Perspectives

For these hypotheses to generate scientific and consequently, clinical value, future research must transition from theoretical plausibility to direct mechanistic and clinical validation. Experimental validation of SR and the RPM within established HBA pathways—including sympathetic and parasympathetic signaling, hormonal axes, and neuroimmune interfaces—is required to determine whether nanotesla-range endogenous cardiac magnetic fields can meaningfully influence neural, autonomic, or molecular processes. Similarly, large-scale multi-center experiments, replicating heart-to-heart synchronization findings are also essential to establish causality and generalizability of evidence supporting geomagnetic and interpersonal interactions as well as HEMF involvement in emotional states and human connection

From a practical standpoint, technological challenges including shielding, noise, cost, and scalability limitations in SQUID, OPM, TMR, and magnetoelectric systems—though currently addressed in several prototype systems—remain significant barriers to scalable clinical translation [23,24,25,26,27,46,47].

Future advances in biosensing technologies along with integration of AI models could allow for a transition from descriptive monitoring to sensor-guided therapeutic modulation. Highlysensitive, miniature and even portable sensors, combined with wearable ECG and HRV-based systems that utilize AI models for signal processing and data synthesis, could eventually enable real-time physiologic and autonomic monitoring, thus opening up the possibility of adaptive neuromodulation strategies where stimulation parameters are continuously adjusted based on ongoing biosignals [20,21,23,25,26,27]. In this framework, magnetic biosignals function not as merely descriptive indicators, but as actionable markers that guide therapeutic interventions (***BioMagnetoTherapies***) and enable personalization of treatments [11,12,13,14].

## 8. Discussion

The incorporation of electromagnetic signaling into the HBA framework extends conventional neurocardiac models beyond neural, hormonal, and inflammatory pathways, introducing an additional measurable biophysical dimension. While the HEMF is technologically validated and theoretically grounded, its extremely weak strength remains the central point of skepticism, requiring careful interpretation within existing biological models.

Across the technologies reviewed —from biosensors to magnetic stimulation—externally inducing, stabilizing or restoring coherence across the HBA where endogenous mechanisms are unable to do so emerges as a shared objective. In this framework, the term BioMagnetoTherapies (BMT) is introduced to distinguish magnetic-based therapeutic strategies oriented towards restoring coherence across the HBA, from conventional pulsed electromagnetic field therapies which act through broader, physiological pathways (angiogenic, anti-inflammatory, stress-protein pathways, etc.). Its defining feature is therefore not merely magnetic exposure, but intentional and strategic modulation of the HBA, with restoration of coherence serving as the central, measurable therapeutic endpoint.

Overall, identifying coherence as a measurable and potentially malleable component of the HBA provides a conceptual bridge between biosensing, environmental modulation, and therapeutic intervention. While caution is warranted given current evidentiary limitations, this framework offers a structured, unifying lens on which future mechanistic clarification and clinical translation may be based.

## Data Availability

No new data were created or analyzed in this study.

## Abbreviations

AI	Artificial Intelligence
HBA	Heart–brain axis
HRV	Heart rate variability
HPA	Hypothalamic pituitary axis
HSP70	Heat shock protein 70
HEMF	Heart’s electromagnetic field
ANS	Autonomic nervous system
EMF	Electromagnetic field
MRI	Magnetic Resonance Imaging
ELFs	Extremely low frequency fields
RPM	Radical Pair Mechanism
SR	Stochastic Resonance
MCG	Magnetocardiography
MS	Multiple Sclerosis
GCNs	Graph convolutional networks
SQUIDs	Superconducting Quantum Interference Devices
SOT	SQUID on tip
RAS	Renin angiotensin system
ECG	Electrocardiogram
EEG	Electroencephalogram
BMT	BioMagnetoTherapies
TMS	Transcranial magnetic stimulation
TMR	Tunneling magnetoresistance
dTMS	Deep transcranial magnetic stimulation
OCD	Obsessive–compulsive disorder
OPMs	Optically pumped magnetometers
MDD	Major depressive disorder
TRD	Treatment-resistant depression
Y-BOCS	Yale-brown obsessive-compulsive score
ECT	Electroconvulsive therapy
LFMS	Low-frequency magnetic stimulation
SMFs	Static magnetic fields
EGFR	Epidermal growth factor receptor
PEMF	Pulsed electromagnetic field
BBB	Blood–brain barrier
PTSD	Post-traumatic stress disorder
RMN	Remote magnetic navigation
